# Comparison of Perioperative Temperature Monitoring Sites During Elective Cesarean Deliveries: A Quality Improvement Initiative

**DOI:** 10.7759/cureus.99890

**Published:** 2025-12-22

**Authors:** Nada Ismaiel, Snehi Shah, Nan Guo, Brendan Carvalho, Kelly Fedoruk

**Affiliations:** 1 Anesthesiology, El Camino Health, Mountain View, USA; 2 Anesthesiology, University of Toledo College of Medicine, Toledo, USA; 3 Anesthesiology, Perioperative and Pain Medicine, Stanford University School of Medicine, Stanford, USA

**Keywords:** cesarean delivery, ob anesthesia, peri-operative medicine, quality improvement, temperature monitoring

## Abstract

Background: Impaired thermoregulation secondary to neuraxial anesthesia during cesarean delivery (CD) can contribute to perioperative hypothermia and maternal morbidity. While temperature monitoring during CDs can detect hypothermia, the optimal temperature measurement modality for CDs under neuraxial anesthesia remains unclear. The aim of this quality improvement (QI) initiative was to compare perioperative temperature measurement modalities during CDs.

Methods: Thirty patients undergoing scheduled CDs were selected for this Institutional Review Board (IRB)-exempt QI initiative. Patients received standard neuraxial anesthesia and intraoperative care including active warming. Temperature measurements were taken at three perioperative time points: at skin incision, within 15 minutes after delivery of the infant(s), and at skin closure. Three modalities were used to measure temperature each time: (1) temperature-sensing Foley catheter, (2) oral temperature probe, and (3) skin temperature probe. Temperature modalities were compared using mean differences ± SD, correlation coefficients, and Bland-Altman (BA) plots.

Results: The mean difference between oral vs. skin and oral vs. bladder temperatures was 0.238°C ± 0.380°C and 0.368°C ± 0.361°C, respectively. The overall temperature correlations between oral vs. skin, oral vs. bladder, and skin vs. bladder were 0.225, 0.588, and 0.198, respectively. In the end, the percentage of hypothermic temperatures (below 36°C) by modality were 0% (oral), 10% (skin), and 26.7% (bladder).

Conclusions: The results suggest a weak correlation between oral and skin temperature measurements, and a moderate correlation between oral and bladder temperatures. These findings should be considered when evaluating hypothermia after CD to enhance patient safety and guide improvement efforts.

## Introduction

Impaired thermoregulation in the context of both general and neuraxial anesthesia for obstetric patients has been linked to perioperative hypothermia and subsequent adverse patient outcomes [1,2]. Perioperative hypothermia is defined as a core temperature less than 36°C [2]. Preventing hypothermia is a key process to improve the safety of surgical care [3]. Hypothermia prevention is especially important during cesarean delivery (CD), where even mild perioperative hypothermia has been associated with adverse maternal and neonatal outcomes. These maternal outcomes include the development of coagulopathies and increased risk for postpartum hemorrhage (PPH), surgical site infections, delayed postpartum recovery, and patient discomfort from shivering [4]. Hypothermia in parturients develops quickly after the administration of neuraxial anesthesia for CD, and recovery of core body temperature after spinal anesthesia takes time, even with active warming [5]. Current literature supports active perioperative warming strategies such as forced air warming and intravenous (IV) fluid warming to mitigate the risk of perioperative hypothermia [4,6].

The optimal perioperative temperature measurement site for awake patients requiring CD under neuraxial anesthesia remains unclear. The classically described gold-standard sites for measuring core body temperature, including measurement at the pulmonary artery, distal esophagus, and nasopharynx [1], are not practical for use in awake patients under neuraxial anesthesia. Oral temperature measurement can be inconvenient, semi-invasive in this setting, and difficult to administer in nauseated or vomiting CD patients. Oral temperature may not be diagnostically reliable relative to true core temperature due to external influences from ambient temperature and probe placement [7]. However, in the awake and alert patient, oral temperature measurement is considered a sufficient estimate of core temperature [8,9]. Bladder temperature measurement represents an alternative site, though some literature suggests it could be less accurate or demonstrate a lag as it is less well-perfused [1]. Temporal skin temperature is a commonly used measurement site during CD, but accuracy in the CD setting is not well established. There is currently limited comparative data between different temperature measurement sites in the setting of CD.

The objective of this quality improvement (QI) initiative was to compare three different perioperative temperature measurement sites: sublingual oral, temporal skin, and bladder thermometry to guide patient care at our center. The primary outcomes were the mean difference between oral and skin temperatures and between oral and bladder temperatures at the end of surgery. We also aimed to determine how measurements varied over the course of scheduled CDs and the incidence of maternal hypothermia at the end of the CD for each of the temperature measurement sites.

## Materials and methods

This report conforms to the SQUIRE 2.0 guidelines for reporting new work to improve healthcare safety [10]. The project was reviewed by the Stanford Institutional Review Board (IRB) and did not meet the definition of human subject research as defined in 45 CFR 46, nor the Federal Drug Administration (FDA) definition of a clinical investigation as defined in 21 CFR 56. Therefore, this QI project was approved for completion without IRB approval.

Setting

This QI project was performed at our institution (Lucile Packard Children’s Hospital at Stanford University). This is an academic level four obstetric center with a CD rate of 32%. We have previously implemented active warming strategies and QI efforts to improve compliance with warming protocols and temperature monitoring. We decided to embark on this QI project to elucidate which monitoring site would be best for our patient population and institution to comply with hospital-wide normothermia efforts. Our current intraoperative temperature monitoring protocol for CDs triggers temporal skin temperature measurements every 15-30 minutes starting after the delivery of the neonate.

Intervention

This was a prospective observational QI evaluation of temperature measurement sites during routine CD care. No randomization was performed. A convenient sample of 30 female patients undergoing scheduled CDs at term gestation at our center was included in this QI effort over a three-month period (May to August of 2023). Our inclusion criteria were all term gestation scheduled cesarean deliveries with neuraxial anesthesia with spinal or combined spinal-epidural (CSE) and planned intraoperative bladder catheterization per standard care. Exclusion criteria included emergent cesarean deliveries, planned or intraoperative conversion to general anesthesia, patient contraindications to neuraxial anesthesia, inability to obtain sublingual oral measurements, absence of bladder catheter placement, and clinician-determined deviation from standard warming protocol. Eligible patients were identified from the scheduled CD list on days when the designated anesthesia provider was available to perform standardized measurements. Consecutive eligible patients on these days were approached until the target sample size was reached. All patients received standard neuraxial anesthesia using a spinal or a CSE technique. Our standardized spinal medication protocol is 0.75% hyperbaric bupivacaine 12 mg with fentanyl 15 mcg and morphine 50-150 mcg. All patients received routine intraoperative care during their CDs as per institutional protocols. This included active warming strategies with IV fluid warming using a 3M™ Ranger fluid warmer model 245 (3M™, St. Paul, MN, USA) and forced air warming using a 3M™ Bair Hugger upper body warming blanket model 52200 with warming unit model 77500 (3M™, St. Paul, MN, USA), as well as maintaining ambient operating room temperature at 22°C. The IV fluid warming was initiated at the start of each CD and continued through the duration of the surgery. Forced air warming was initiated from the time of infant(s) delivery until the end of the CD, or earlier to accommodate patient preference and comfort if required.

Measurements

**Figure 1:**
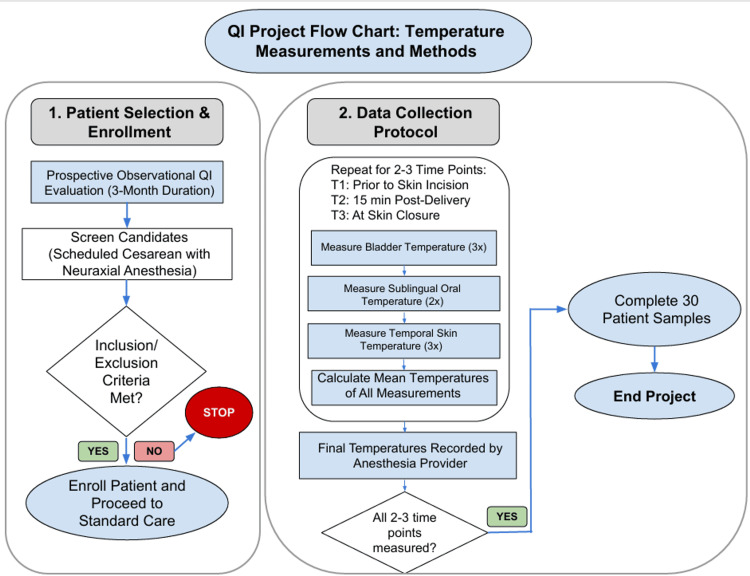
Methodology for comparison of perioperative temperature monitoring sites during scheduled cesarean deliveries

Temperature measurements were taken at three perioperative time points: prior to skin incision, within 15 minutes after delivery of the infant(s), and at skin closure (Figure 1). Three sites were used to measure temperature at each time point: (1) a temperature-sensing bladder catheter (BARD model A119216M SureStep Lubri-Sil 16F Temperature Sensing Foley Catheter with Hydrogel) (BARD Medical Division, Covington, GA, USA), (2) a sublingual oral temperature probe (Welch Allyn model SureTemp Plus Model 690 electronic thermometer) (Welch Allyn, Inc., Skaneateles, NY, USA), and (3) a temporal skin temperature probe (TAT-5000 temporal artery thermometer) (Exergen Corporation, Watertown, MA, USA).

At each perioperative time point, three bladder measurements, three temporal skin measurements, and two sublingual oral temperature measurements were each taken approximately one minute apart. The mean of each bladder, temporal skin, and sublingual oral measurements was used as the final temperature at each time point for each site for the analysis. Only two sublingual oral temperature measurements were taken at each time point (versus three as used for the other sites) to minimize patient discomfort. The operating room (ambient) temperature was also recorded at each time point (prior to skin incision, within 15 minutes after delivery of the infant(s), and at skin closure). At each time point, bladder temperatures were recorded directly from the patient monitor as the bladder catheter temperature probe was connected to the patient’s vital signs monitor. The sublingual oral temperatures were collected by placing the probe in the sublingual space (calibration accuracy 0.1°C at 36.0°C). Temporal skin temperatures were measured by placing the probe at the forehead and sliding across to the region of the right or left temporal artery, as per manufacturer instructions. All temperatures were measured and recorded in degrees Celsius (°C). All measurements were taken and recorded by the same single anesthesia provider for all the CDs to minimize measurement errors and maintain consistent methodology.

Analysis

Statistical analysis was completed using STATA 14.0 (StataCorp., College Station, TX, USA). The primary outcomes were the mean difference between oral and skin temperatures and oral and bladder temperatures at the end of surgery. We used a convenient sample size for this QI initiative. Based on prior research conducted at our institution to determine the efficacy of active warming during CD, this sample size would provide adequate power for our primary aim. Temperature sites were compared using mean ± SD differences at each perioperative time point. Sublingual oral temperature was used as the site to represent near-core body temperature [1,8,9,11] and the site against which temporal skin and bladder temperatures were compared. Correlations between the three temperature sites were plotted and calculated. Bland-Altman (BA) plots were constructed to compare skin and bladder measurements to sublingual oral temperature readings. The incidence of maternal hypothermia was calculated at the end of every CD for each temperature site.

Patient and public involvement

This QI initiative did not directly involve patients or the public in the study design, research question development, choice of outcome measures, or recruitment. As a technical evaluation of temperature measurement methods during standard clinical care, patients received routine perioperative care per institutional protocols. The project was motivated by our commitment to improving maternal safety during CD, which aligns with patient-centered care priorities. Temperature monitoring directly impacts patient comfort and safety by helping prevent hypothermia-related complications. Results will be disseminated through institutional channels to improve our temperature monitoring protocols based on the findings.

## Results

The demographic data for the patient population is summarized in Table 1, including procedure duration (from skin incision to skin closure, in minutes) and quantitative blood loss (QBL, in mL). Out of the 30 patients enrolled in this QI initiative, two patients experienced a PPH. One patient experienced a PPH due to extension of the hysterotomy incision and did not require second-line uterotonic therapy. The second patient experienced a PPH due to uterine atony and received two second-line uterotonics, a 1 g dose of tranexamic acid, and placement of an intrauterine vacuum device for mechanical control of the PPH. None of the 30 patients included in this QI initiative required any transfusion of blood products.

**Table 1:** Demographic data for patient population (N = 30) IQR: interquartile range; CD: cesarean delivery; CSE: combined spinal-epidural; QBL: quantitative blood loss

Demographics	N = 30
Age; median (IQR)	30 (31 to 40)
Ethnicity; N (%)	
Asian	10 (33.3%)
Caucasian	11 (36.7%)
Hispanic	7 (23.3%)
Other	2 (6.7%)
BMI (kg/m^2^); median (IQR)	31.0 (26.2 to 33.8)
Parity; N (%)	
0	8 (26.7%)
1	13 (43.3%)
>2	9 (30.0%)
Gestational age (weeks); median (IQR)	39 (38.4 to 39.1)
Prior CD; N (%)	7 (23.3%)
0	11 (36.7%)
1	12 (40.0%)
>2	7 (23.3%)
Anesthetic technique; N (%)	
CSE	10 (33.3%)
Spinal	20 (66.7%)
QBL (mL); median (IQR)	718 (522 to 851)
Duration of procedure (min); median (IQR)	52.5 (42 to 59)

**Figure 2:**
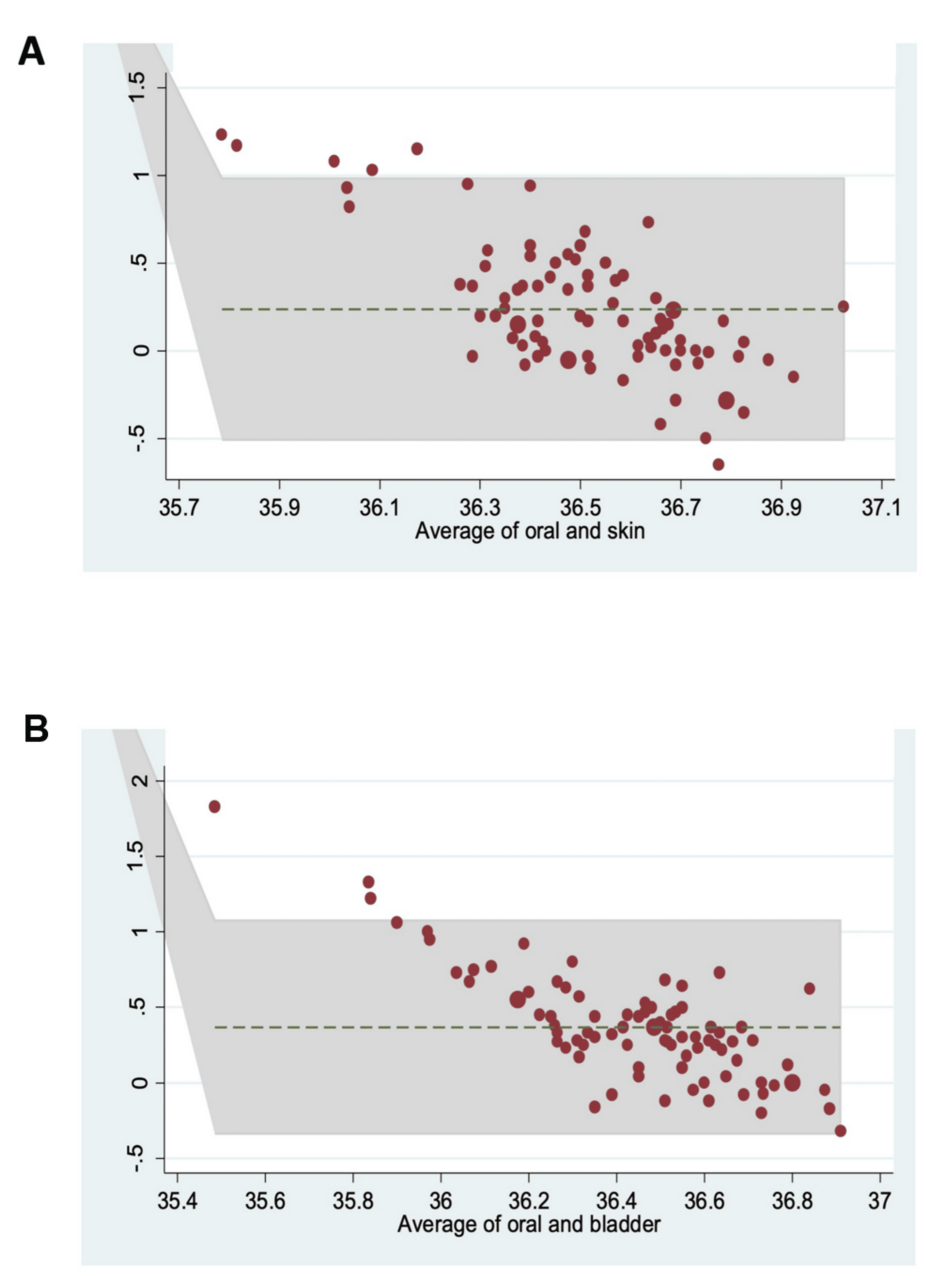
Bland-Altman difference plots comparing oral and skin temperatures (A) and oral and bladder temperatures (B). For (A), 6.98% of measurements were outside the 95% limits of agreement (-0.507 to 0.984), and average temperatures were between 35.78°C and 37.02°C. For (B), 6.49% of measurements were outside the 95% limits of agreement (-0.338 to 1.075), and average temperatures were between 35.48°C and 36.91°C

The mean difference between sublingual oral and temporal skin temperatures was 0.238°C ± 0.380°C (95% limits of agreement -0.507 to 0.984). The mean difference between sublingual oral and bladder temperatures was 0.368°C ± 0.361°C (95% limits of agreement -0.338 to 1.075). These findings are illustrated in BA difference plots in Figure 2.

**Figure 3:**
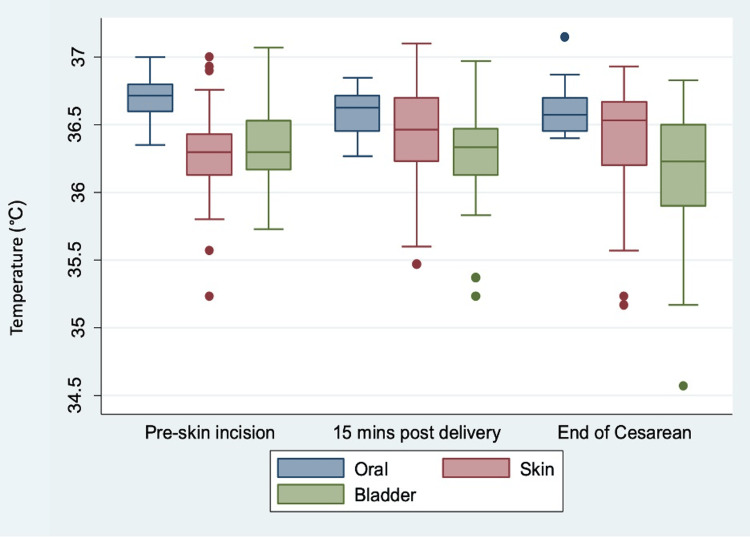
Box-and-whisker plots of oral, skin, and bladder temperatures at three perioperative time points (pre-skin incision, 15 minutes post delivery, and at the end of the cesarean delivery)

Sublingual oral, temporal skin, and bladder temperatures at all three perioperative time points are illustrated in Figure 3. The overall correlation coefficient (with 95% confidence intervals) between sublingual oral and temporal skin temperatures was 0.225, sublingual oral and bladder temperatures was 0.588, and temporal skin and bladder temperatures was 0.198 (Table 2).

**Table 2:** Spearman correlations (with 95% confidence intervals) at each perioperative time point and overall correlations between oral, skin, and bladder temperatures

Perioperative time point	Oral and skin temperatures	Oral and bladder temperatures	Skin and bladder temperatures
Pre-skin incision	0.135 (-0.245, 0.542)	0.444 (0.076, 0.719)	0.194 (-0.189, 0.577)
15 min post delivery	0.174 (-0.201, 0.521)	0.604 (0.314, 0.894)	0.002 (-0.417, 0.415)
Skin closure	0.522 (0.233, 0.764)	0.710 (0.504, 0.894)	0.439 (0.109, 0.727)
Overall	0.225 (0.018, 0.431)	0.588 (0.444, 0.721)	0.198 (-0.006, 0.403)

The percentage of maternal hypothermia (below 36°C) by site at surgical end was 0% for sublingual oral temperature (N = 0/30), 10% for temporal skin temperature (N = 3/30), and 26.7% for bladder temperature (N = 8/30).

## Discussion

In this QI initiative, we compared three different temperature monitoring sites (sublingual oral, temporal skin, and bladder) to determine how the current site (temporal) that we use during CDs at our center compares. Our results show significant differences in temperature measurements based on the site used. Only sublingual oral and bladder temperatures consistently showed moderate and strong correlations. Importantly, the incidence of hypothermia at the surgical end varied significantly based on the measurement site. These findings are important to consider, as intraoperative maternal temperature measurement during CD guides active warming strategies to prevent hypothermia-associated maternal morbidity and impacts treatment strategies when hypothermia is encountered.

Guidelines emphasize that intraoperative temperature monitoring during CDs is important [3,12,13]. Numerous studies outside of obstetric anesthesia have investigated various temperature measurement sites, evaluating their efficacy in accurately measuring temperature, correlation with gold-standard core temperature monitoring methods, and comparative performance with one another [14-20]. Measuring temperature in awake parturients undergoing CD is particularly nuanced because special attention is required for patient comfort and avoidance of invasive measurement strategies, potential artifact from patient shivering or shaking, difficult-to-perform oral measurements in nauseated or vomiting patients often encountered during CD, and the potential impact on skin-to-skin bonding between the patient and neonate.

Oral temperature has previously been used as a reliable measure of core temperature [1,8,9,11]. Sublingual oral temperature was the site taken as the closest to core temperature in this QI initiative. While more recent literature has demonstrated that oral temperature may not be a true reflection of core or near-core temperature [7], a recent study by Mah et al. used the same sublingual oral thermometer as was used in this QI initiative to represent the gold standard to which other sites were compared with reasonable accuracy [11]. However, oral temperatures are more invasive than skin measurements, are not practical in nauseated or vomiting patients, and interrupt skin-to-skin bonding between the patient and neonate. Due to these limitations, we elected to use temporal skin measurements at our institution. However, the accuracy of this site has been questioned.

A study of neurosurgical patients demonstrated that temporal artery skin thermometry does not provide accurate measurements to serve as a surrogate for core temperature in awake or asleep patients intra- or postoperatively [14]. Several studies have similarly suggested that temporal artery skin temperature devices can be inaccurate and inconsistent in reflecting core or near-core body temperature [15-18]. In a study comparing temporal artery skin temperature to true core temperature using a pulmonary artery catheter (PAC) in intensive care patients, there was up to 25% variability between temporal skin temperatures and core temperatures using a PAC [21]. Some factors contributing to this inaccuracy may be related to diaphoresis, environmental air flow, and vasopressor use that may have limited temporal artery blood flow and, therefore, led to a lower temperature measured at that skin site [18]. While temporal skin and bladder temperatures correlate poorly [14], temporal skin temperatures have been shown to correlate well with oral temperatures preoperatively and postoperatively [15]. Studies comparing temperature measurement sites in the CD setting are lacking.

In this QI initiative, we demonstrated a weak correlation between both temporal skin and sublingual oral temperatures and temporal skin and bladder temperatures (correlation coefficients 0.225 and 0.198, respectively). We see this reflected in our BA plot, where 6.98% of measurements were outside the 95% limits of agreement. This suggests that, while fast and convenient to obtain, temporal artery skin temperatures are not reliably comparable to sublingual oral (which we used as a surrogate for near-core temperature) methods of measurement.

Measurement of temperature via bladder thermometry is attractive for CDs because it is non-invasive (for CD patients who have a bladder catheter placed after neuraxial anesthesia) and can provide continuous, real-time temperature monitoring that does not require interruption of the anesthesiologist’s workflow. Bladder thermometry is believed to be a reliable surrogate of core temperature when compared to pulmonary artery temperature using a PAC [22]. A recent study successfully demonstrated a lower incidence of maternal hypothermia during CD with active and passive warming strategies using a temperature-sensing bladder catheter [23]. These authors concluded that bladder thermometry, which was taken to reflect core temperature [20], is indeed a superior measure when compared to oral (sublingual) and temporal artery skin temperatures, and comparable in accuracy to tympanic membrane temperatures [14,24]. There is skepticism in using bladder thermometry to reflect core temperature due to the low perfusion of the bladder and its proximity to the open surgical field during CD [1], thus causing a potential lag in temperature measurement [1,5]. However, this may be offset by increased renal blood flow and a high urine flow state, which occurs during pregnancy and CD [20]. Our QI initiative demonstrated a moderate correlation overall between bladder and sublingual oral temperatures (correlation coefficient 0.588), with the correlation becoming progressively stronger over time during the CD, and only 3.49% of measurements outside the 95% limits of agreement.

Unlike bladder and temporal skin temperatures, sublingual oral temperatures showed the smallest decrease during CD. This likely reflects an excellent response to active warming strategies with IV fluid, forced air, and operating room temperature maintenance. Sublingual oral temperatures showed the narrowest interquartile ranges of temperatures compared to those of bladder and temporal skin temperatures at all perioperative time points. Taken together, these findings suggest some increased reliability of sublingual oral thermometry, similar to previous literature [8,9,11].

Measuring maternal temperature to detect hypothermia in patients undergoing CD is important in guiding active warming strategies. A recent study estimated the incidence of maternal perioperative hypothermia (below 36°C) to be 32.5% for patients undergoing scheduled CD under spinal anesthesia without any active warming [23]. In our QI initiative, however, we demonstrated the incidence of maternal hypothermia of 0% when measured with sublingual oral thermometry at the end of the CD with active warming. This is compared to an incidence of hypothermia of 10% when measured via temporal skin thermometry and 26.7% with bladder thermometry. These findings should be considered when evaluating hypothermia after CD, and when comparing hypothermia incidences between institutions that use different temperature measurement sites.

Our QI initiative had several limitations. Since all patients were awake under neuraxial anesthesia and invasive core temperature monitoring with an esophageal probe or PAC was not possible, a true core temperature control to which all sites can be compared could not be established. We utilized a convenient sample size for this QI project, and the sample size was not sufficient to determine differences in patient outcomes. Only one of our sites (bladder thermometry) offered continuous intraoperative temperature monitoring, while sublingual oral and temporal skin thermometry only offered intermittent measurement limited to the selected perioperative time points. We limited our comparison to three commonly used sites (sublingual oral, temporal skin, and bladder). Including more novel non-invasive temperature sites such as tympanic thermometry, zero-heat flux thermometry, and an ingestible telemetric sensor could have offered additional informative temperature comparisons that may more accurately measure maternal temperature during CD [5,24-26]. Future directions should explore the use of these newer sites, especially since all these sites offer continuous measurements during the entire CD and are non-invasive, yielding more comfort for awake parturients.

## Conclusions

The goal of this QI initiative was to improve maternal temperature monitoring practices at our institution. We sought to better understand how our available thermometry sites compare to one another, and how our use of these sites compares to the currently available literature. The optimal temperature measurement device in this setting is not established, and accuracy and patient comfort during CD need to be considered. Our results show significant differences in temperature measurements based on the sites used. Only sublingual oral and bladder temperatures consistently showed moderate and strong correlations. The incidence of hypothermia at surgical end varied significantly by measurement site. These findings should be considered when evaluating hypothermia after CD to guide improvement efforts and when comparing hypothermia incidences between institutions.
